# The Effect of Cold Swaging of Tungsten Heavy Alloy with the Composition W91-6Ni-3Co on the Mechanical Properties

**DOI:** 10.3390/ma14237300

**Published:** 2021-11-29

**Authors:** Paweł Skoczylas, Olgierd Goroch, Zbigniew Gulbinowicz, Andrzej Penkul

**Affiliations:** Department of Mechanics and Weaponry Technology, Faculty of Mechanical and Industrial Engineering, Warsaw University of Technology, Narbutta 85, 02-524 Warsaw, Poland; pawel.skoczylas@pw.edu.pl (P.S.); zbigniew.gulbinowicz@pw.edu.pl (Z.G.); andrzej.penkul@pw.edu.pl (A.P.)

**Keywords:** tungsten heavy alloys, liquid phase sintering, mechanical properties, kinetic energy penetrator

## Abstract

The paper presents the results of studies on the effects of heat treatment and cold-work parameters on the mechanical properties and microstructure of the tungsten heavy alloy (WHA) with the composition W91-6Ni-3Co. Tungsten heavy alloy (WHA) is used in conditions where strength, high density, and weight are required. The material for testing as rod-shaped samples was produced by the method of powder metallurgy and sintering with the participation of the liquid phase and then subjected to heat treatment and cold swaging. The study compares the effect of degree deformation on the strength, hardness, microhardness, and microstructure of WHA rods. The conducted tests showed that heat treatment and cold-work allowed to gradually increase the strength parameters, i.e., tensile strength $\sigma_{uts}$, yield strength $\sigma_{ys}$, elongation ε, hardness, and microhardness. These processes made it possible to increase the tensile strength by over 800 MPa (from the initial 600 MPa after sintering to the final value of over 1470 MPa after heat treatment with cold swaging deformation with reduction of 30%) and the hardness from 32 to 46 HRC.

## 1. Introduction

Tungsten heavy alloys (WHA) are high-density materials combined with high strength, plasticity, and toughness. WHA is used in various industries. Most commonly, WHA is used in applications requiring counterweights, inertial masses, radiation shielding, and ordnance products, as well as a number of other applications [1,2]. Due to the very high melting point, these alloys are usually made from a mixture of powders, which are sintered at a liquid phase [3,4] at about 1520 °C after compaction. At this temperature, nickel and cobalt melt and combine with tungsten grains. After sintering, the material can be described as a two-phase composite consisting of spherical tungsten grains embedded in a bonding phase that is a Ni/Co/W alloy [5,6,7,8,9,10,11,12,13].

WHA rods obtained after the sintering process involving the liquid phase and additional vacuum heat treatment (dehydrating) are characterized by the following physical properties: tensile strength $\sigma_{uts}$ = 800–1100 MPa, yield strength $\sigma_{ys}$ = 750–800 MPa, elongation ε = 20–35%, and HRC hardness 26–32 [14,15]. Tungsten heavy alloy is used in conditions where strength, high density, and weight are required. In this section, it is discussed in detail where and why tungsten heavy alloy (WHA) is used. For some applications (especially military), the strength of such materials is too low. For many years, the investigations have been carried out in the world to increase the penetrating capabilities of kinetic energy projectiles. The depth of penetration for such projectiles depends most of all on the dynamic hardness and the density of projectile’s material, unit mass of the projectile, and on the hit velocity. In order to obtain possibly high values, the penetrators are usually made from: tungsten heavy alloys (WHA) with density ρ ≈ 17,500 kg/m^3^ and depleted uranium DU with density ρ ≈ 19,000 kg/m^3^ [16,17].

APFSDS (armor-piercing fin stabilized discarding sabot) ammunition developed at the end of the sixties for smooth bore guns was a breakthrough in questions referring to the tank armor penetration depth. A significant increase in armor penetration depth (against the projectiles of former generation) was provided by the possibilities for reaching muzzle velocities up to 1800 m/s for the smooth gun APFSDS projectiles and for obtaining the penetrator’s slenderness (L/d) above 10. The steel with high mechanical performance was used first for the penetrators. In the next step, APFSDS projectiles with carbide-tungsten cores were developed. The development of sintering technology (deployment of such additives as nickel, iron, cobalt, copper, and rhenium) and the mechanical processing of tungsten sinters (e.g., perimeter swaging) made the slenderness of the penetrator increase from 10 to over 30 [18,19,20,21].

Recent research on tungsten heavy alloys used for subcaliber bullet cores has focused on increasing their effectiveness, as measured by the thickness of the armor pierced by them. Such actions increase strength while maintaining the highest possible density [16,17,18,19,20,21]. The desire to increase the strength properties while maintaining the possible high density, which, depending on the tungsten content in the alloy, has a negative impact on the resistance to dynamic loads. In this work, the findings of measurements of the hardness and microhardnesses of tungsten grains and matrix were described in the cross-sections of the rod, depending on the swaging deformation of cold-work processing.

## 2. Materials and Methods

**Figure 1:**
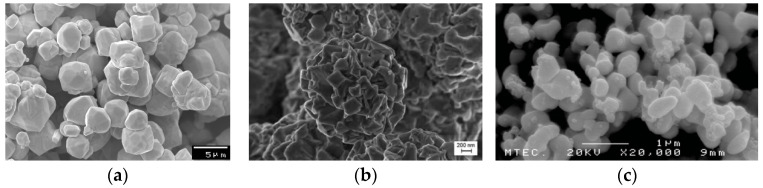
The morphology of the used powders. (**a**) Tungsten, (**b**) nickel, (**c**) cobalt.

Liquid-phase sintering is a sintering in which the liquid phase coexists with the solid-phase particles. This method makes it possible to obtain material with a density close to or almost equal to the theoretical density. The liquid phase is formed during sintering as a result of the eutectic reaction of the metals used as additives with tungsten. Nickel, used as the basic alloy component forming the binding phase (matrix) of WHA alloys, lowers the sintering temperature and ensures the good wettability of tungsten grains and its solubility in the liquid phase. The other ingredients, i.e., Fe, Cu, Co, and others, function to lower the sintering temperature, reduce the solubility of tungsten in nickel, improve the mechanical properties of the matrix, and increase the strength at the interface between the tungsten grain and the matrix phase [22]. In order to reduce the oxides, the sintering process is carried out under a hydrogen atmosphere. A heavy tungsten alloy with the composition 91%wt W, 6%wt Ni, 3%wt Co was chosen as a base alloy, to evaluate the effect of post sintering mechanical treatment, on the mechanical and structure properties of this alloy. The characteristics of the elemental powders are given in Table 1, and the morphologies of the different powders are illustrated in Figure 1. 

**Figure 2:**
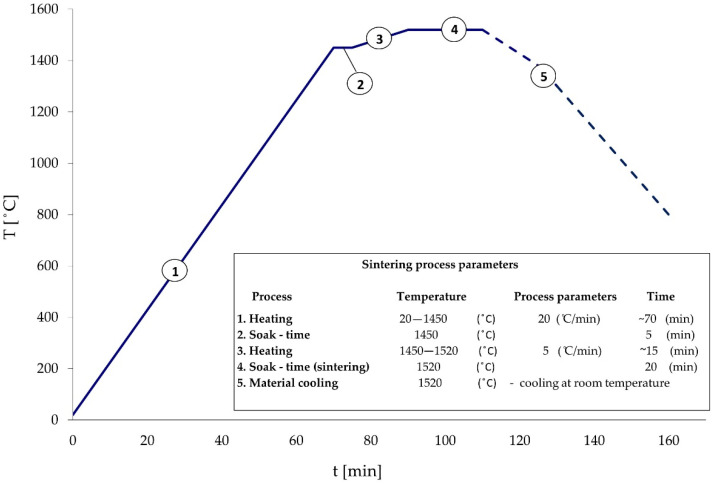
Sintering temperature diagram.

The first step in the production of tungsten heavy alloys was to mix the appropriate amount of powders to homogenize the blends in a drum mixer. For the selected chemical composition of WHA, the theoretical density was 17.46 g/cm^3^. The mixing time was based on previous studies [23,24] and was 20 h. The next stage was pressing in a steel die, into which about 7 kg of the powder mixture was poured each time. Pressing was carried out on a Voeller angle press under a pressure of 200 MPa. Rod-shaped moldings with a diameter of approximately 42 mm and a length of 510 mm were obtained. After the moldings were placed on the Al_2_O_3_ ballast on a molybdenum tray, they were placed in a Vacuum Industries chamber furnace. Sintering was carried out in a hydrogen atmosphere according to the assumed cycle (Figure 2). The sintering with the liquid phase took place for about 20 min at the temperature of 1520 °C with the liquid phase [25].

After sintering, the rods were heat-treated. The aim was to make the alloy ductile so that it could be cold-worked. The heat treatment was carried out in a vacuum furnace under a reduced pressure of 5 Pa in the temperature range of 950–1150 °C. After annealing, the total time of which was 9 h, and the rods were cooled rapidly [26]. The annealing temperature and time were selected based on previous tests [27,28,29,30]. 

In the next stage of the manufacturing process, the WHA rods were cold-worked. Before the swaging process, the rods were machined. The aim was to ensure uniform cold swaging deformation on the rod cross-section. The cold swaging deformations of the rods [31,32] was carried out on a STEYER SPH06.09 four-lever swaging machine with reductions in the range of 15–30%. The following values of swaging deformation were obtained in the range: 15% in one pass and 20–30% in two passes.

**Figure 3:**
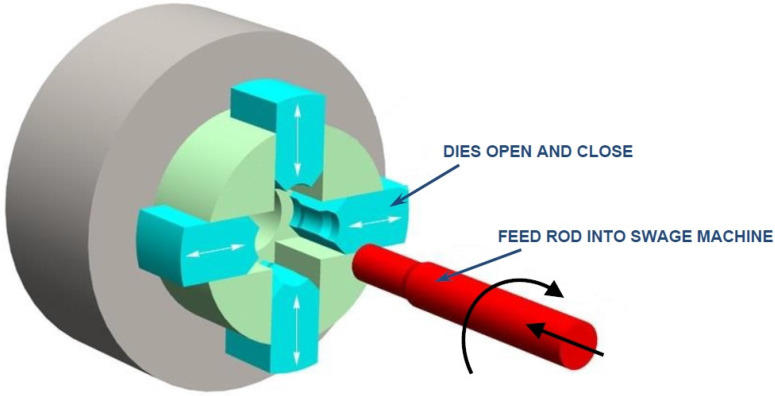
Graphic illustration of the tungsten heavy alloys cold swaging process.

The deformation of the material takes place in the oscillating swaging machines in the plane passing through the rod axis (Figure 3). Lever-attached dies obtain the oscillatory motion of the eccentrics. This results in a sinusoidal approximation of the speed of moving the die working. The opposite arrangement of the dies internally balances the system of forces so that the swaging machine does not transfer loads to the foundation. Cold swaging may be carried out either simultaneously with four dies or by alternating the counter-rotating dies in pairs.

The deformation process is stable when the friction angle is greater than the tangent of half the angle of inclination β of working surfaces.

**Figure 4:**
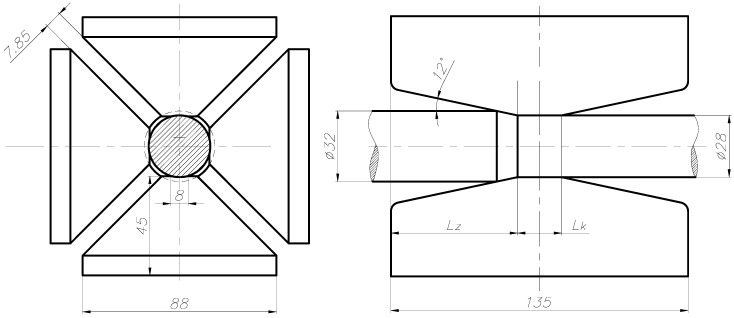
Diagram of the rod cold swaging process.

The dies for swaging heavy tungsten alloys are made of NC10 steel. These dies have a converging crushing zone (Lz length) at an angle of 12° and a calibration segment (Lk length) measured along the die axis (Figure 4).

**Figure 5:**
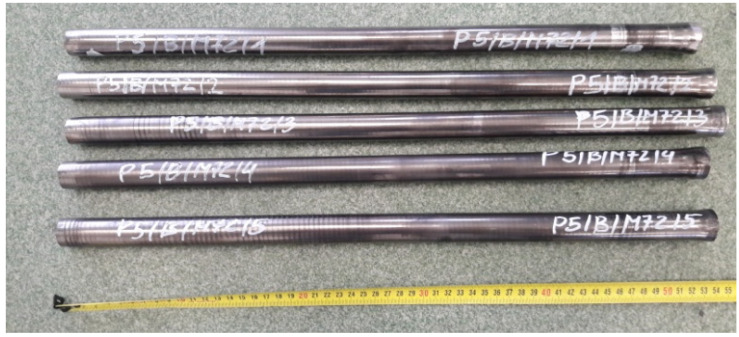
View of rods samples (WHA) after cold swaging.

The rods were swaged at the following parameters: feed 1.5 mm/s and revolutions 30 rpm (Figure 5). The rods were swaged at two aisles. The cold swaging deformation was calculated from the formula:

(1) $$deformation\;value=\frac{{\left(outer\;diameter\right)}^{2}-{\left(inner\;diameter\right)}^{2}}{{\left(outer\;diameter\right)}^{2}}\times 100\%$$

**Figure 6:**
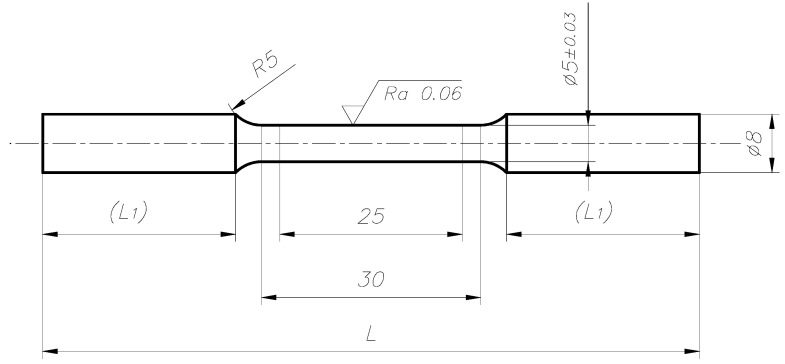
Dimensions (in mm) of samples intended for mechanical tensile tests (L—~95 mm, L_1_—~30 mm).

The length of the swaged rods was 520–540 mm, and the diameter was 26–29 mm. The results obtained depended of the cold swaging deformation value and the initial length of the rod. Hardness, microhardness, and microstructure observations were performed for the materials thus obtained, and tests of mechanical properties were carried out using a static tensile test [33,34]. The aim of the static tensile test was to determine: tensile strength $\sigma_{uts}$, yield strength $\sigma_{ys}$, elongation ε. The tests were carried out on 5-fold round samples with machining in the axis of the rod with a nominal measuring diameter of 5 mm (Figure 6). The test samples were prepared in accordance with the PN-EU 10002-1:2004 standard. The tensile test was carried out on an INSTRON model 1115 with a strain rate of 6.6 × 10^−4^ s^−1^.

Observations on the metallographic microscope were carried out perpendicular and parallel to the axis of the rod. The observations of the microstructure were carried out on the Nikon Eclipse MA-200 Microscope (Nikon Corporation, Tokyo, Japan). The metallographic microsections of the rod sections were made by hot-mounting. The metallographic samples were ground on SiC sandpaper in the gradation range 320–1200 and then polished with a diamond suspension. Grinding and polishing of the samples were performed on a Shapir 520 semiautomatic grinder–polisher. The hardness and microhardness tests were carried out on transverse microsections. The measurement was carried out in the middle part of the sample to reflect the place where strength samples were taken. Microhardness studies were conducted on the microhardness tester machine (Future-Tech FM 810, Future-Tech Corp. Tokyo, Japan) under a load of 25 g and a dwell time of 15 s. Spacing was about 4 diagonals of the microindenter. The average obtained value was based on a sample of 20–25 measurements in a given area. The hardness test was performed with a standard load on an HRC hardness tester machine (HR-150A, Jinan Hensgrand Instrument Co., LTD., Jinan, China).

## 3. Results

### 3.1. Test Results in a Static Tensile Test

**Figure 7:**
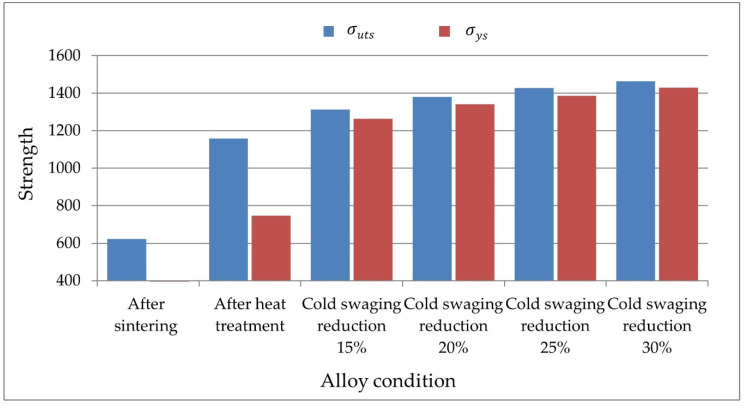
The results of tests of tensile strength $\sigma_{uts}$ and proof stress $\sigma_{ys}$.

**Figure 8:**
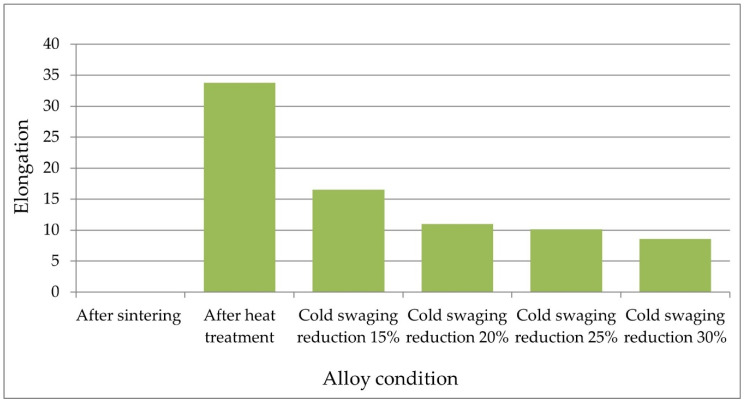
Elongation test results.

The test specimens were prepared according to Figure 6 and taken from the rod axis at a distance of about 100 mm from the edge. The static tensile test was carried out with a strain rate of 6.6 × 10^−4^ s^−1^. The results of the tests of mechanical properties are presented below in tabular form (Table 2) and in the graphs (Figure 7 and Figure 8). 

The material is brittle immediately after sintering. The samples are broken in the elastic range. The obtained tensile strength is 622 MPa. The use of heat treatment increases the tensile strength to 1150 MPa, the proof strength to 750 MPa, and the elongation to 34%. The heat treatment and the cold swaging process strengthens the material in the following values:

For cold swaged with a reduction of 15%, the tensile strength increases to 1313 MPa, the yield point increases to 1263 MPa, and the elongation decreases to 16.5%.

The increase in the reduction of cold swaged deformation to 20% increased the strength by 68 MPa to the value 1380 MPa, the yield strength by 25 MPa to the value 1268 MPa, and the decrease in elongation by 5% to the value 11%.

The increase in the reduction of cold swaged deformation to 25% causes a further increase in the strength to the value of 1428 MPa, the yield strength to 1386 MPa, and the decrease in elongation to 10%.

For the highest reduction of cold swaged 30%, the highest strength parameters were obtained:$\;\sigma_{uts}$ = 1464 MPa, $\sigma_{ys}$ = 1430 MPa, and the lowest elongation value ε = 8.6%.

### 3.2. Analysis of the Microstructure

**Figure 9:**
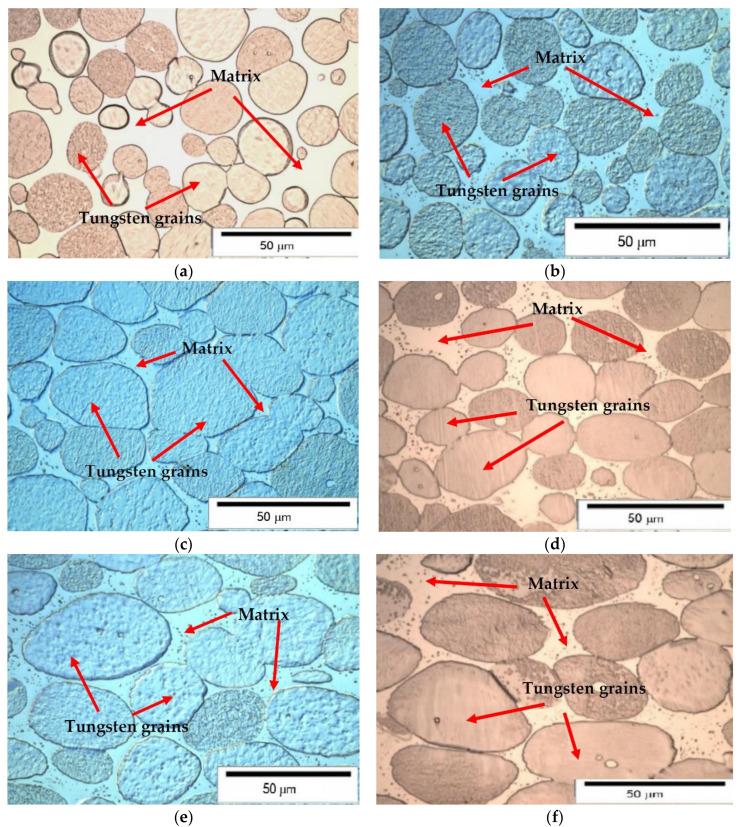
Microstructure of PR200 alloy after sintering (**a**), after heat treatment (**b**), and after cold swaging deformation with reduction of: 15% (**c**), 20% (**d**), 25% (**e**), and 30% (**f**).

Figure 9 contains metallographic [35] photographs showing the microstructure of 91WNi6Co3 alloy: after sintering (a), after heat treatment (b), and after cold swaging deformation with reduction of 15% (c), 20% (d), 25% (e), and 30% (f). After heat treatment [36] in the matrix, during the observation of the microstructure, small separations are visible, invisible in the matrix after sintering. In the microstructure of the alloy, after cold swaging (Figure 9b), tungsten grains are visible, deformed plastically along the direction of swaging.

As a result of observation of metallographic samples after sintering and heat treatment, it can be concluded that the rods are homogeneous throughout the cross-section [37]. Tungsten grains in the microstructure are correctly formed, and no pores or discontinuities were observed.

### 3.3. Microhardness Test Results

The investigated material is two-phase. It consists of tungsten grains and a W/Ni/Co matrix. For this reason, the microhardness of tungsten grains and matrix was tested separately. 

**Figure 10:**
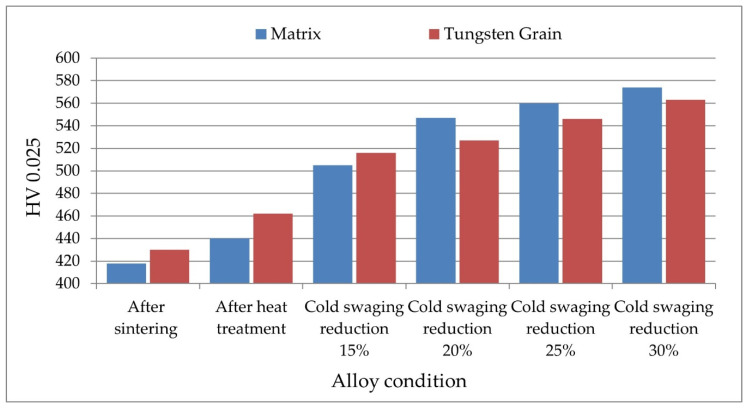
The results of microhardness HV0.025 of tungsten grains and matrix measured along the rod axis.

**Figure 11:**
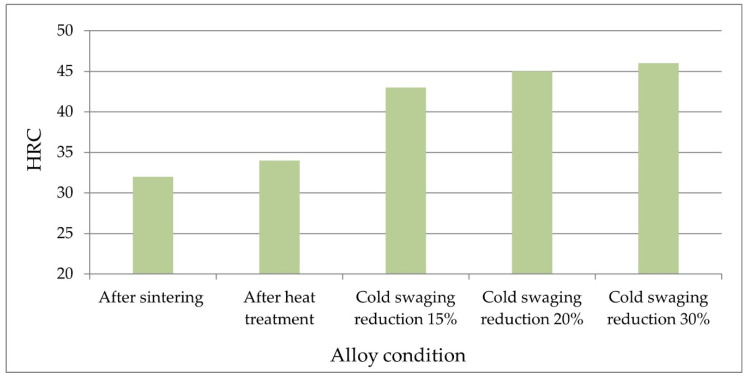
The results of the HRC hardness of the tungsten grains and the matrix measured along the bar axis.

**Figure 12:**
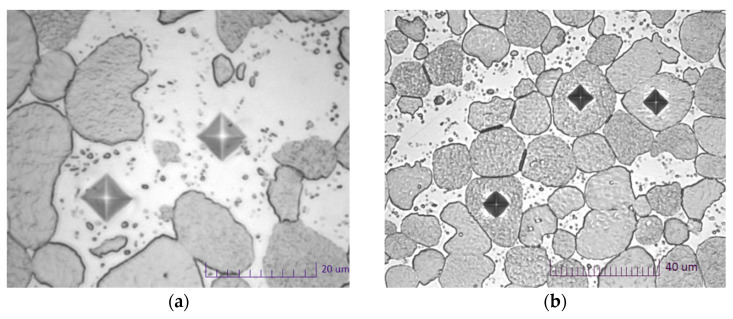
View of indenter imprints in the matrix and tungsten grains (with lens magnification: (**a**)—×100, (**b**)—×50).

Table 3 and Figure 10 and Figure 11 show the results of measurements of hardness and microhardness of rods after heat treatment (input condition) and rods for various deformation degrees of cold swaging. Figure 12 shows the method of taking the imprints of the micro-indenter in the matrix and of the tungsten grains.

The results of the studies on the effect of the degree of cold swaging deformation on the strength parameters were presented at work [25]. The current work focuses on examining the effect of the degree of swaging on the microhardening on the WHA and on determining the differences in the microhardness of tungsten grains and matrix. This determines the minimum value of the cold swaging deformation degree, ensuring a homogeneous hardening of the microstructure components, with a known rod diameter.

The presented test results show that the applied machining cycle (heat treatment, then swagging the material according to a different value of deformation) causes a gradual increase in the hardness and microhardness of the alloy. The microhardness of tungsten grains in the material immediately after sintering is 430 HV0.025, while the matrix is 418 HV0.025.

Heat treatment increases the microhardness of tungsten grains and matrix by 20–30 HV0.025 units (tungsten grains 460 HV0.025 and matrix 440 HV0.025).

Cold swaged with a reduction of 15% [38] increases the microhardness by about 60–70 HV0.025 units in relation to the condition after hot treatment. The microhardness of tungsten grains is 516 HV0.025 units, and of matrix, 505 HV0.025 units. For a higher value of reductions (20%, 25%, and 30%), the microhardness of the matrix is higher than the microhardness of tungsten grains. Increasing the deformation value in cold swaging to 20% causes a greater strengthening of the matrix than of tungsten grains. The matrix microhardness is 547 HV 0.025 (increase by 40 units in relation to the cold swaging deformation value of 15%). The microhardness of tungsten grains is 527 HV 0.025 (an increase of 10 HV 0.025 units compared to the previous state). Cold swaged with a reduction of 25% causes further strengthening of the matrix and tungsten grains. The degree of microhardness increase for tungsten grains is higher than the degree of strengthening the matrix. The matrix microhardness is 560 HV 0.025 (increase by 13 units in relation to the cold swaging deformation value of 20%). Microhardness of tungsten grains is 546 HV 0.025 (increase by 20 HV 0.025 units in relation to the cold swaging deformation value of 20%). Cold swaged with a reduction of 30% in relation to the 25% increases the matrix microhardness by a further 14 HV 0.025 units and amounts to 574 HV 0.025. The microhardness of tungsten grains in the rod axis for cold swaging reduction of 30% is higher by 17 units (with reference to 25%) and amounts to 563 HV0.025. The presented results show that for reduction value of 20% and 30%, the matrix strengthening is higher than the hardening of the tungsten grains.

The results of the Rockwell hardness measurements show that the initial hardness after sintering is 32 HRC. After heat treatment, it is increased to 34 HRC. The greatest increase in hardness occurs at the initial cold swaging with reduction 15% and amounts to 43 HRC. Further swaging the material allows one to obtain a hardness of 45–46 HRC. The same as for HRC microhardness increases after heat treatment and with increasing degree of swagging. However, it reaches maximum values with a swaging deformation of about 30%. Above this value, the hardness does not change.

## 4. Discussion

The measurements of the alloy [39,40] composition 91W-6NI-3Co after the sintering process showed that the real alloy density is equal to the theoretical alloy density (ρ = 17.46 g/cm^3^). This means that the alloy produced by powder metallurgy and sintering with the participation of the liquid phase is a nonporous material. This is evidenced by the analysis of the microstructure of the metallographic specimens longitudinal and transverse to the axis of the sintered rod. It showed that the quality of the material was obtained, in which no material defects in the volume of the material in the form of pores, microshrinkage, or voids were found. The metallographic analysis also showed that the alloy microstructure shows the correct two-phase structure consisting of ellipsoidal tungsten grains in the matrix of the binding phase. 

The applied heat treatment causes precipitations in the area of the binding phase of the alloy, with probably intermetallic phases with an increased tungsten content. Precipitations in the area of the binding phase (supersaturation) are probably one of the main reasons for the increase in strength and microhardness of the matrix after heat treatment.

**Figure 13:**
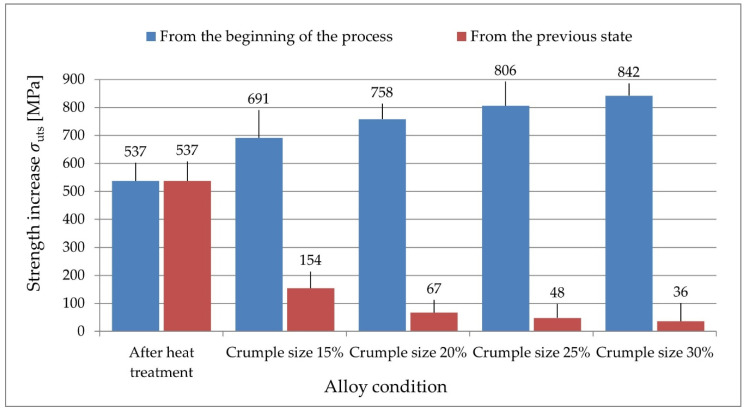
Increase in tensile strength for different WHA states.

The diagram (Figure 13) shows the difference in the increase in tensile strength both from the initial state (after sintering) and from the previous state.

The presented results show that the highest increase in strength occurs after heat treatment (increase by nearly 540 MPa) and after applying 15% of swaging deformation (increase by 690 MPa from the sintering state and by 150 MPa from the state after heat treatment). The higher deformation of cold swaging, the smaller the increase in tensile strength. With an increase in swaging deformation value from 15% to 20%, the increase in strength is nearly 70 MPa. For the value of 25%, the increase in strength is close to 50 MPa, and for the final value 30%, the increase in strength is close to 40 MPa.

The total increase in strength that was obtained using heat-treatment and cold-work processes from the initial state (after sintering) to the final state (after 30% deformation value) is over 840 MPa. The strengthening created during the plastic working process in the material causes a decrease in plasticity by 25% (from approximately 34% after heat treatment to 9% after working 30%).

It should be noted that the applied heat treatment for WHA materials is a process that increases two opposing parameters: tensile strength $\sigma_{uts}$, yield strength $\sigma_{ys}$, and elongation ε.

Rotary cold swaging is an incremental metalworking process for reducing the cross-sectional area or otherwise changing the shape of rods, tubes, or wires by repeated radial blows with two or more dies. This article discusses the applicability of swaging and metal flow during swaging. Therefore, with a thorough insight into the material flow, it could be understood how to control it in order to achieve desired properties. Furthermore, by applying this method, different local cold hardening could be achieved by different total strain. It is necessary to make elongated subcaliber projectiles [16,17,18,19,20,21], which are classified as kinetic artillery (KE) projectiles characterized by high kinetic energy related to the area unit of the penetrator cross-section. The depth of penetration of kinetic artillery shells into metallic armored partitions depends on the following factors: the dynamic hardness and density of the shell materials (in this case the core). 

The strengthening due to the cold swagging processing is evident and was expected. Due to intensive strain shear bands being formed in and across the grains, the diameter reduction and intensive straining resulted in thinning and shearing of the surface layer. The grain size was not evaluated as well, although due to the appearance of the shear bands, the fine microstructure can be expected.

Metallographic investigation revealed that microstructure after sintering and heat treatment consists of elongated grains with a moderate amount of fine recrystallized grains. The elongated grains are stretched during rotary swaging.

## 5. Conclusions

Based on the research, it can be concluded that: 

The applied parameters of the sintering process make it possible to obtain a homogeneous porous material with the actual density equal to the theoretical density. This material has a two-phase microstructure, consisting of spherical tungsten grains evenly distributed in the matrix of the binding phase.

The applied parameters of heat treatment make it possible to change the properties of the WHA material from brittle to elastic-plastic, which are the basis for cold swagging.

The cold swaging deformations with reductions of 15–20% is the optimal value causing the highest increase in the strength properties of WHA material.

For the initial 15% of deformation value, the highest increase in WHA microhardness (both matrix and tungsten grains) can be observed.

An increase cold swaging deformations with reductions of 25% or 30% results in a poorly visible increase in tensile strength. Instead, it causes a significant reduction in plasticity.

For cold swaging deformations with reductions above 20%, there was no increase in hardness using the Rockwell method.

The highest increase in WHA microhardness occurs for the initial 15% of cold swaging deformations.

The use of heat and plastic treatments allows one to obtain a material with increased mechanical properties. The obtained strength and plastic values allow the use of this material for the production of modern kinetic penetrators.

In the swaging process, the degree of the microhardening of the matrix in the material is higher than the degree of the tungsten grains.

## Figures and Tables

**Table 1:** Characteristics of the elemental powders.

Powder	W	Ni	Co
Shape	polygonal	sponge	nearly spherical
Particle size (µm)	1–4	3–7	5.0–6.5
App density (g/cm^3^)	4.3	1.6–2.6	0.8–1
Melting temperature (°C)	3422	1455	1495
Density (g/cm^3^)	19.3	8.9	8.9

**Table 2:** WHA mechanical properties test results.

Condition of Alloy	Tensile Strength $\sigma_{uts}$ [MPa]	Proof Stress $\sigma_{ys}$ [MPa]	Elongation ε [%]
After sintering	622 ± 6	did not occur	0
After heat treatment	1159 ± 4	747 ± 5	33.8 ± 0.9
Cold swaging reduction 15%	1313 ± 5	1263 ± 5	16.5 ± 1.1
Cold swaging reduction 20%	1380 ± 6	1342 ± 17	11.0 ± 1.4
Cold swaging reduction 25%	1428 ± 4	1386 ± 8	10.1 ± 1.3
Cold swaging reduction 30%	1464 ± 13	1430 ± 18	8.6 ± 1.4

**Table 3:** Results of measurements of microhardness HV 0.025 and hardness HRC.

Machining Status	Matrix [HV 0.025]	Tungsten Grain [HV 0.025]	Hardness [HRC]
After sintering	418 ± 21	430 ± 16	32
After heat treatment	440 ± 14	462 ± 18	34
Cold swaging reduction (15%)	505 ± 28	516 ± 24	43
Cold swaging reduction (20%)	547 ± 26	527 ± 17	45
Cold swaging reduction (25%)	560 ± 27	546 ± 23	45
Cold swaging reduction (30%)	574 ± 29	563 ± 18	46

## Data Availability

The raw/processed data required to reproduce these findings cannot be shared at this time as the data also forms part of an ongoing study.
